# Gender differences in advanced activities of daily living: evidence from the longitudinal study of health and aging in Mexico 2012–2018

**DOI:** 10.3389/fragi.2025.1544493

**Published:** 2025-07-28

**Authors:** Martha A. Sánchez-Rodríguez, Mariano Zacarías-Flores, Lesly Estefanía Castañeda-Sánchez, Víctor Manuel Mendoza-Núñez

**Affiliations:** ^1^ Research Unit on Gerontology, Zaragoza Faculty of Higher Studies, National Autonomous University of Mexico, Mexico City, Mexico; ^2^ Division of Obstetrics and Gynecology, Gustavo Baz Prada Hospital, Institute of Health of the State of Mexico, Nezahualcóyotl, Mexico

**Keywords:** advanced activities of daily living, gender, healthy aging, functional capacity, MHAS, AADL

## Abstract

**Background:**

Performing advanced activities of daily living (AADLs) is a component of healthy aging (HA) because it involves functional capacity. The ability to perform them can be hampered by several factors, which appear different for men and women.

**Objective:**

To evaluate the performance data of AADLs in older Mexican adults from Mexican Health and Aging Study (MHAS) from 2012 to 2018 and to determine the risk factors for not performing AADLs.

**Methods:**

A secondary longitudinal analysis of the 2012 and 2018 waves of the MHAS was conducted. Adults ≥60 years, from both sexes, who answered at least eight of the nine questions analyzed, without or only mild cognition impairment in 2012, and who were interviewed in both waves were included. An AADL construct with nine questions from the MHAS including physical/leisure, social and productive domains was used. The Cox proportional regression model was used as a longitudinal analysis to determine the risk factors to not perform ≥3 AADLs.

**Results:**

4,738 adults were ≥60 years old and met the inclusion criteria, 2,617 were women (54%). Total AADLs were diminished in 2018 (2.68 ± 1.39 vs. 2.61 ± 1.34, p < 0.01); however, women performed more AADLs in 2018 than in 2012, contrary to men. Risk factor to not perform ≥3 AADLs in women were age ≥70 years and sedentary lifestyle. Men have the same risk factors in addition to low scholarship and live in urban locations. After control by confounder factors, the risk of not performing ≥3 AADLs was in the overall model HR = 1.25 (95% confidence intervals (CI): 1.17–1.37), women HR = 1.20 (95%CI: 1.08–1.32), and men HR = 1.26 (95%CI: 1.17–1.35).

**Conclusion:**

Our findings show that the execution of ≥3 AADLs is age-dependent over 80 years. Although this capacity could be gender-dependent, the environment and public policies can be determining factors.

## 1 Introduction

Aging is a gradual and adaptive process because of a relative decrease in biological responses and reserve for recovering or maintaining homeostasis, which is caused by genetics and the wear and tear accumulated from the challenges faced by the personal history in each environment (Mendoza-Núñez et al., 2016). Aging is individualized, with physical, psychological and social changes that are not necessarily in concordance with chronological age.

In 1999, the World Health Organization (WHO) defined “active aging” as “the process of optimizing opportunities for health, participation and security in order to improve the quality of life as people age”, with active participation being the key element to maintain health, manage social security and achieve greater well-being, for which it is essential that older adults maintain physical independence, cognitive capacity and autonomy (World Health Organization, 2002). Likewise, the WHO (2015) specified the concept of healthy aging (HA) defined as “the process of developing and maintaining the functional ability that enables well-being in older age”, specifying that functional capacity is made up of “the intrinsic capacity of the individual, relevant environmental characteristics and the interactions between the individual and these characteristics”, specifying that intrinsic capacity (IC) is “the composite of all the physical and mental capacities of an individual”, determined by genetic inheritance, health and personal characteristics (World Health Organization, 2015). In this sense, the assessment of IC considers psychological and sensory aspects, vitality, cognition and locomotion, as well as mobility, learning and decision-making capacity, social relationships and contribution to society (Beyen et al., 2024; Behr et al., 2023).

In this framework, physical and cognitive functional capacity can be assessed at the community level through the performance of advanced activities of daily living (AADLs), which consider not only the daily personal activities of survival independence, but also those that allow social and economic independence and autonomy in other daily areas such as work activities, social participation, personal development, leisure and recreation (Moreira de Oliveira et al., 2015; Cornelis et al., 2019; Rao et al., 2021; Nascimento et al., 2022). For this reason, we propose that the measurement of AADLs constitutes a good indicator of HA in community cohort studies such as the Mexican Health and Aging Study (MHAS), since AADLs could be considered as the outcome of physical and cognitive functional capacity and the favorable environment, and implicitly we could infer that they are linked to psychological well-being.

A fundamental variable to consider in the evaluation of AADLs is sex and gender, since biologically, women are more vulnerable than men to the prevalence and incidence of some chronic non-communicable diseases (NCDs), although their longevity is greater (Fritz et al., 2024). However, the environment may favor their functional and cognitive capacity, evaluated through AADLs, considering that many community gerontological programs have a feminist orientation and the participation of men is very limited, which could constitute a gerontological social advantage for women.

Concerning AADLs, we previously validated and probed a construct to evaluate them with the 2018-Mexican Health and Aging Study (MHAS) database (Sánchez-Rodríguez et al., 2023), a national longitudinal survey of community-dwelling Mexican adults aged 50 years and older from urban/rural areas with follow-up interviews in 2001, 2003, 2012, 2015, 2018 and 2021 (Mexican Health and Aging Study, 2025). The use of national databases to conduct studies to probe different constructs and indices, such as IC (Pan et al., 2024; Zhang et al., 2024; Gutiérrez-Robledo et al., 2021) or HA (McLaughlin et al., 2020; Gómez et al., 2021; Torres et al., 2023), is ideal due to large sample sizes. However, most of these studies focus on the decline and diseases during aging. Our study was the first to include individuals without cognitive impairment, and the findings show that Mexican adults aged 60 years or more have the risk of not performing ≥3 AADLs when age increases, with low schooling, if they reside in urban areas and/or have a sedentary lifestyle and have at least a comorbidity, however, it was a cross-sectional study, which precludes any inference of causal relationships (Sánchez-Rodríguez et al., 2023). Therefore, this study aimed to evaluate the performance of AADLs of older Mexican adult participants of the MHAS from 2012 to 2018 in a longitudinal analysis, and to determine the risk factors for not performing ≥3 AADLs to confirm our previous findings.

## 2 Materials and methods

### 2.1 Study description

A secondary longitudinal analysis from 2012 (15,723 respondents) and 2018 (17,114 subjects) waves of the MHAS was conducted (MHAS Mexican Health and Aging Study, 2015). As described above, in the MHAS, adults of 50 years and older from rural and urban areas with national representation were interviewed in 2001 with 2003, 2012, 2015, 2018, 2021 and 2024 follow-up interviews. To refresh the sample, new samples of adults with the original inclusion criteria were added in 2012 and 2018. The aim of the survey was to obtain information to examine the aging process, the disease and disability related, and socioeconomic aspects to prospectively evaluate disease impact on the health, function, and mortality of older adults in Mexico. The survey includes questions about demographics, health in multiple domains (chronic conditions, functionality, depression, cognition), socioeconomic conditions, income, family background, time spent occupied and psychosocial issues, among others (Mexican Health and Aging Study, 2025; Inegi, 2018; Wong et al., 2017). All respondents are identified by a key to their home address to maintain anonymity. Data is available, after registration, from the webpage http://www.mhasweb.org/. We selected the 2012 and 2018 waves because not all questions used to evaluate AADLs were included in the survey of previous waves. At the moment of this analysis, only 2001 to 2018 waves were available.

**FIGURE 1:**
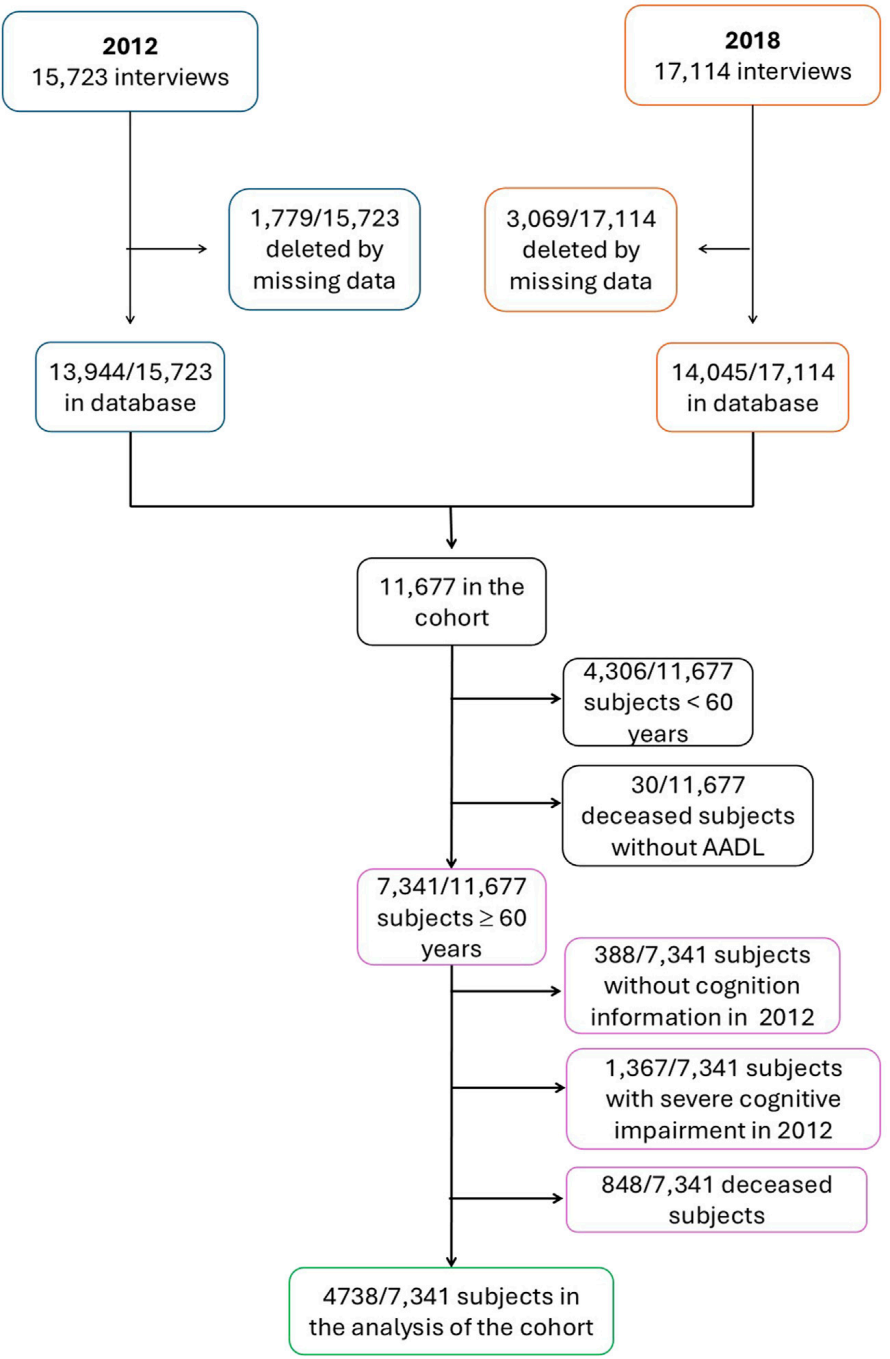
Flowchart of eligible data from MHAS 2012 and 2018 waves and final study cohort.

In this study, the inclusion criteria were adults aged 60 years and older, both sexes, who answered at least 8 of the 9 questions analyzed, and without or with mild cognition impairment in 2012. All the subjects who meet the inclusion criteria and who were interviewed in both waves were included in the cohort sub-sample (4,738 respondents). Moreover, the deceased persons between the two waves were considered as contributing to the cohort if they met the requirement of answering the questions of AADL in 2012 (848 elders) (Figure 1).

### 2.2 AADLs

An AADL construct included nine questions of the MHAS. These incorporated physical/leisure, social and productive domains (three questions each); these activities were selected from previously validated questionnaires (Moreira de Oliveira et al., 2015; Días et al., 2015; Dias et al., 2019). The answers must be scored in binary fashion as 0 (no) or 1 (yes) if the older adult conducted the corresponding activity; then, the total of the activities performed is computed. A performance deficit occurs if the older adult scores <3 AADLs. This construct shows a reliability of 60% (p < 0.0001) with Kuder-Richardson 20 (KR-20) test obtained from older adults aged 60 years and over without cognitive impairment to avoid misclassification (Sánchez-Rodríguez et al., 2023).

Questions form the MHAS included in the AADL construct are in the physical/leisure domain (i.e., “Assist with a sport or social club?”; “Assist in a lecture, seminar, or class?”; “Sew, embroider, knit or do other crafts?”), social domain (i.e., “Do you attend religious services?”; “Talk to relatives or friends on the phone with or use the computer to send email or use the internet?”; “Did you take care of a sick family member?”), and the productive domain (i.e., “In the last 2 years, did you participate in any volunteer work for a religious, educational, charity organization or the community?“; “Work as a volunteer or help with a non-profit organization without pay or compensation?“; “During the last year, did you have a primary paid job?“) (Supplementary Table S1).

### 2.3 Sociodemographic, lifestyle, and health conditions data

Potential confounding variables were included in the study and were separated into sociodemographic, lifestyle and health conditions variables. The sociodemographic variables were sex, age group, residence place (rural <2,500 inhabitants and urban >2,500 inhabitants) INEGI (2024a), educational level (illiterate, elementary (6 years), middle school (9 years), high school (12 years) and college or higher (schooling in university)), and marital status (married/in a civil union, never married, divorced/separated, widowed). The lifestyle variables included whether they were currently smoking cigarettes, drinking alcohol, and being sedentary (no exercise or no hard physical work >3 times per week). For health conditions we considered if the older adults had any NCD such as diabetes mellitus, arterial hypertension or stroke; other NCDs were not included because they were infrequent in the cohort population. We considered cognitive impairment if the interviewee did not know the complete date and could not recall at least three words in an immediate word recall test (Gutiérrez-Robledo et al., 2021), and it was classified as without, mild or severe impairment. Although sarcopenic obesity increases with age and can modify the ability to carry out AADLs, the survey does not have parameters to establish this condition, thus in this work only it was only considered overweight/obesity as confounder variable, which was determined by the body mass index obtained by dividing weight (in kilograms) by squared height (in meters), using 25.0 kg/m^2^ as a cut-off in accordance with international guidelines (Jensen et al., 2013).

### 2.4 Statistical analysis

We calculate means and standard deviations or standard error from the quantitative data, and frequencies, percentages, and 95% confidence intervals (CI) from the categorical data. Kolgomorov-Smirnov test was used to establish the normality of the quantitative data. Because the normal distribution was not verified, for independent comparison we use Mann-Whitney U test with quantitative data and chi-squared test for categorical data, and the dependent comparison was conducted with Wilcoxon signed-rank test and McNemar’s test, respectively. Both comparisons were stratified by gender and age group (<75 years and ≥75 years). The <75 years cut-off was used because life expectancy in Mexico in 2012 and 2018 was 74.9 years (INEGI, 2024b). Moreover, the age was categorized into three groups: 60–69 years, 70–79 years and ≥80 years.

To assess the consistency of the construct, we obtained a random sample of 100 participants without cognitive impairment from the cohort base for each year. KR-20 was calculated as a reliability test obtaining moderate results, as in the original validation (Sánchez-Rodríguez et al., 2023), for the 2012 subsample KR-20 = 0.60 and for the 2018 subsample KR-20 = 0.61.

The Cox proportional regression model was used for longitudinal analysis. The total of months between interviews (2012 and 2018) of each older adult individual was used as a time variable, besides the months of the deceased subjects from the interview in 2012 to their death. Several models were constructed using sociodemographic (sex, age group, place of residence, educational level and marital status), lifestyle (smoking, alcohol intake and being sedentary) and health condition (NCDs, cognitive and nutritional status) data as independent variables. For dependent variables, we calculated the difference of dichotomous AADLs (<3 AADLs and ≥3 AADLs) between both years, and we acknowledge the risk if the older adult did not perform <3 AADLs after 6 years (Sánchez-Rodríguez et al., 2023). Age group, marital status and cognition state in 2018, and educational level, were included as ordinal variables. Also, we obtained the difference between 2018 and 2012 status of smoking cigarettes, drinking alcohol, being sedentary, being obese or with overweight and the presence of NCD as dichotomous scale (Yes/No). It was considered as risk if the respondent had the habit/condition in at least one of the years or in both.

Only the variables that showed statistical significance in their correspondent Cox regressions (sociodemographic, lifestyle, and health condition) were included in the final models, which were both overall and gender stratified. All the models were built with hazard-proportion assumptions. Control categories include the 60–69-year age group, female gender, rural residence, married/in-a-civil union, with college or higher education, and without cognitive impairment. Risk factor was considered if the hazard ratio (HR) > 1 and the 95% CI did not include the 1.0 values.

We created a forest plot to represent risk factors associated with performing <3 AADLs, and a Kaplan-Meier survival plot stratified by gender and age group. A two-tailed p-value lower than 0.05 was considered statistically significant. The data were processed using the software packages SPSS V.25.0 (IBM SPSS Statistics, Armonk, NY, United States) and Microsoft Excel 365.

## 3 Results

### 3.1 Characteristics of the analyzed data

In the MHAS 2012 and 2018 databases, 15,723 and 17,114 adults were included. In the 6-year cohort, 11,667 adults remained but only 7,341 (63%) respondents have 60 years and over, of which 4,738 (65%) adults met the inclusion criteria, being 2,617 women (54%). Between 2012 and 2018, 848 (12%) elders died and 5% (388/7,341) lacked the information to assess cognition (Figure 1).

More than half of the population in the cohort were from the 70–79 years age group (2,511, 53%). Most older adults had elementary schooling (2,729, 58%), and they were mainly married or in a civil union (2,842, 60%); moreover, 3,885 (82%) lived in an urban location. Men were with partners, while women remained mostly unaccompanied (78% vs 55%, p < 0.0001). In addition, although they more frequently engage in physical activity more than 3 times a week (710, 33%), men had more unhealthy habits, mainly smoking, and were obese in a high proportion (1,326, 63%). Women were more likely to be diagnosed with arterial hypertension (63% vs 46%, p < 0.0001), diabetes mellitus (31% vs 25%, p < 0.0001), and mild cognitive impairment (31% vs. 28%, p = 0.025) than men (Table 1).

**TABLE 1:** Sociodemographic characteristics, lifestyle habits, health conditions, and a total of activities advanced of daily living performed by the total population of 2012–2018 MHAS cohort and stratified by gender.

Characteristic	Total (n = 4,738)	Men (n = 2,121, 46%)	Women (n = 2,617, 54%)
Age (years) in 2012	68.7 ± 6.6	68.9 ± 6.7	68.5 ± 6.6^a^
Age (years) in 2018	74.7 ± 6.6^b^	75.0 ± 6.7	74.5 ± 6.6^c^
Age group in 2018 60–69 years 70–79 years ≥80 years	1,185 (25%, 24%–26%) 2,511 (53%, 52%–54%) 1,042 (22%, 21%–23%)	509 (24%, 22%–25%) 1,389 (54%, 53%–55%) 566 (22%, 21%–23%)	784 (26%, 25%–27%) 1,597 (53%, 53%–55%) 633 (21%, 20%–22%)
Years of education	5.4 ± 4.4	6.0 ± 4.9	4.9 ± 4.0^d^
Educational level^a^ Illiterate Elementary Middle school High school College or higher	758 (16%, 15%–17%) 2,729 (58%, 57%–59%) 711 (15%, 14%–16%) 189 (4%, 3%–5%) 378 (8%, 7%–9%)	318 (15%, 14%–16%) 1,182 (56%, 55%–57%) 275 (13%, 12%–14%) 108 (5%, 4%–6%) 248 (12%, 11%–13%)	440 (17%, 16%–18%) 1,547 (59%, 58%–60%)^e^ 436 (17%, 16%–17%)^f^ 81 (3%, 2%–3%)^f^ 130 (5%, 4%–6%)^g^
Residence place Urban (Population ≥2,500 inhab.) Rural (Population <2,500 inhab.)	3,885 (82%, 81%–83%) 853 (18%, 17%–19%)	1,675 (79%, 78%–80%) 446 (21%, 20%–22%)	2,210 (85%, 84%–86%)^g^ 407 (15%, 14%–16%)
Marital status in 2018 Married/in a civil union Never married Divorced/separated Widowed	2,842 (60%, 59%–61%) 190 (4%, 3%–5%) 332 (7%, 6%–8%) 1,374 (29%, 28%–30%)	1,654 (78%, 77%–79%) 51 (3%, 2%–3%) 112 (5%, 4%–6%) 299 (14%, 13%–15%)	1,188 (45%, 44%–46%)^g^ 140 (6%, 5%–7%)^g^ 220 (8%, 7%–9%)^g^ 1,075 (41%, 39%–42%)^g^
Lifestyle habits Smoking	1,837 (39%, 38%–40%)	1,306 (62%, 61%–63%)	521 (20%, 19%–21%)^g^
Alcohol intake	1,046 (22%, 21%–23%)	700 (33%, 32%–34%)	346 (13%, 12%–14%)^g^
Physical activity (>3 times/week)	1,232 (26%, 25%–27%)	710 (33%, 32%–34%)	522 (20%, 19%–21%)^g^
Health condition Diabetes mellitus Arterial hypertension Stroke Obesity (BMI >25 kg/m^2^) Mild cognitive impairment^h^ Severe cognitive impairment^h^	1,332 (28%, 27%–69%) 2,625 (55%, 54%–56%) 116 (2%, 1%–3%) 2,843 (60%, 59%–61%) 1,421 (30%,29%–31%) 474 (10%, 9%–11%)	527 (25%, 24%–26%) 981 (46%, 45%–47%) 60 (3%, 2%–4%) 1,326 (63%, 62%–64%) 604 (28%, 27%–29%) 212 (10%, 9%–11%)	805 (31%, 30%–32%)^g^ 1,644 (63%, 62%–64%)^g^ 56 (2%, 1%–3%)^e^ 1,517 (58%, 57%–59%)^f^ 817 (31%, 30%–32%)^e^ 262 (10%, 9%–11%)

Wilcoxon signed-rank test between years.

^a^ p < 0.0001. Mann-Whitney U test between genders.

^b^ p < 0.0001. Wilcoxon signed-rank test between years.

^c^ p < 0.01.

^d^ p < 0.0001.

^e^ p < 0.05. Chi-squared test between genders.

^f^ p < 0.001.

^g^ p < 0.0001.

^h^ 2018 wave data. Quantitative data are means ± standard deviation, categorical data are frequencies (%, 95% confidence intervals [95%CI]).

### 3.2 AADLs in 2012 and 2018

**FIGURE 2:**
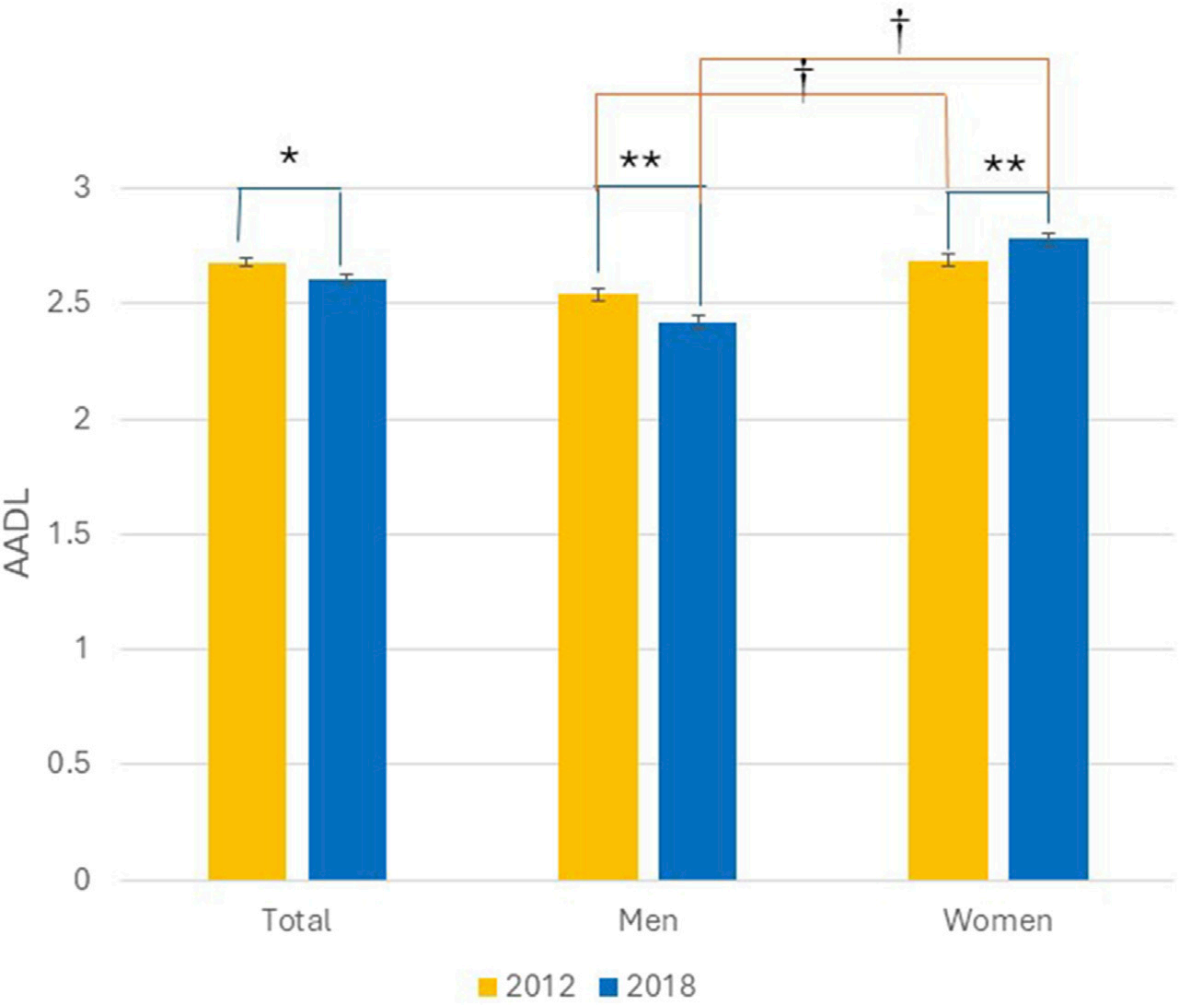
Total of advanced activities of daily living in 2012 and 2018. Total population and stratified by gender. Bars represent means and error bars the standard error. Wilcoxon signed-rank test between years *p < 0.01, **p < 0.0001. Mann-Whitney U test between genders ^†^p < 0.0001. From 2012 and 2018 MHAS data.

Total AADLs were diminished in 2018 (2.68 ± 1.39 vs. 2.61 ± 1.34, mean difference p = 0.002); however, women performed more AADLs in 2018 than in 2012 contrary to men, who performed fewer activities. Notably, those women always performed more AADLs than men (Figure 2).

**FIGURE 3:**
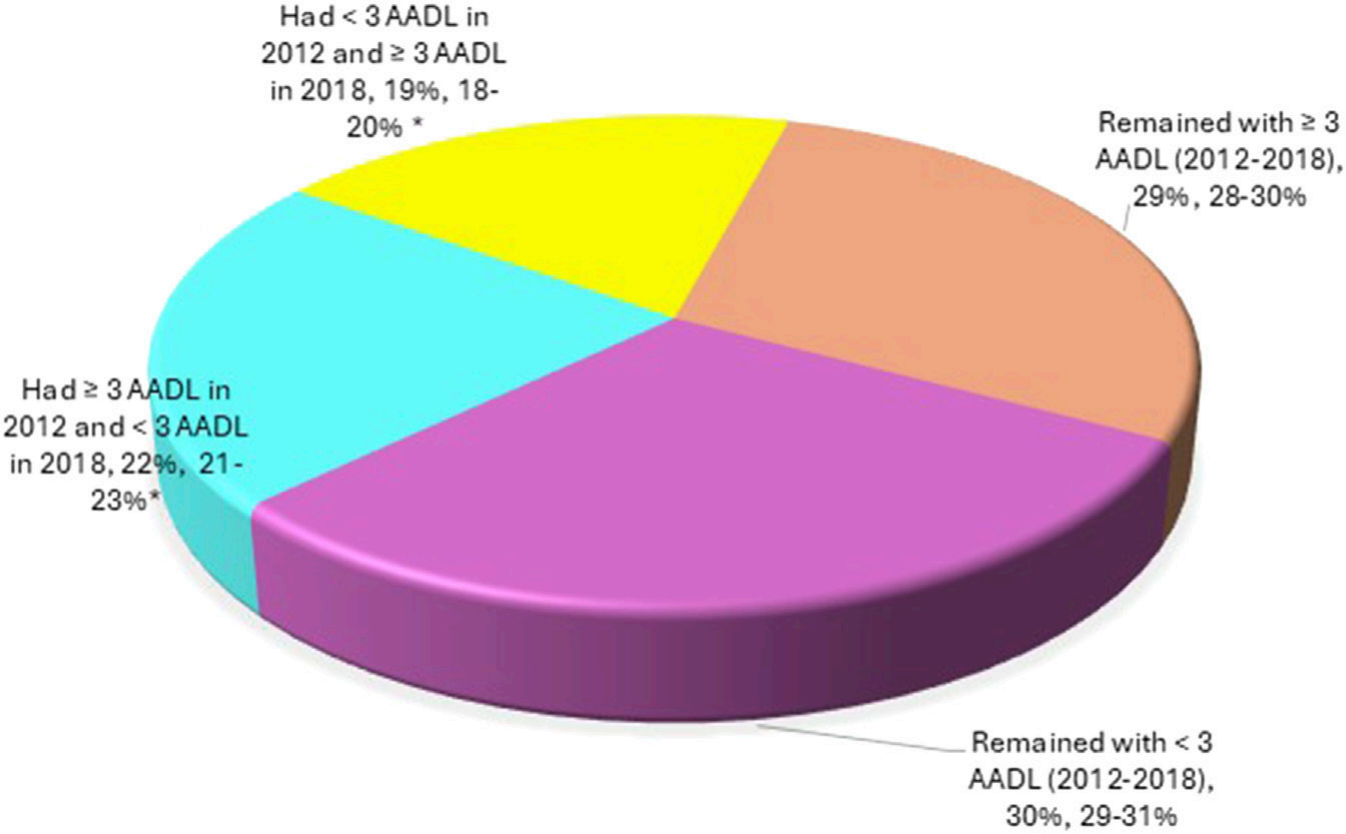
Percentage of AADLs performed by older adults in 2018 compared to 2012. Data show percentage and 95% confidence intervals. Chi-squared test, *p < 0.0001; subjects remained with ≥3 AADLs as control.

Nearly 60% of older adults did not make any changes to their AADLs. In both years, 29% performed ≥3 activities and 30% did not complete more than 3 activities. Notably, 19% of the older adults did not perform 3 or more AADLs in 2012, but in 2018 they already did (Figure 3).

In 2018, older adults decreased their attendance at academic classes and their participation in craft activities but increased their contact with family and friends via phone or internet, as well as taking paid work. In the overall assess, 1,386/2,616 (53%) women and 891/2,121 (42%) men carried out ≥3 AADLs in 2018 (proportion difference p < 0.0001) (Table 2).

**TABLE 2:** Prevalence of advanced activities of daily living by year. From 2012 to 2018 MHAS data.

Activity	2012 (n = 5,586)	2018 (n = 4,737)
Attending a sports or social club	393 (7%, 6%–8%)	275 (6%, 5%–7%)^a^
Attend a lecture, seminar, or class	816 (15%, 14%–16%)	403 (9%, 8%–10%)^b^
Sew, embroider, knit or other crafts	1,703 (31%, 30%–32%)	1,147 (24%, 23%–25%)^b^
Attend religious services	4,615 (83%, 82%–84%)	3,790 (80%, 79%–81%)^b^
Talk to relatives or friends on the phone or use the internetetc.	3,899 (70%, 69%–71%)	3,705 (78%, 77%–79%)^b^
Take care of a sick family member	911 (16%, 15%–17%)	829 (18%, 17%–19%)
Participate in any volunteer work in community	966 (17%, 16%–18%)	777 (16%, 15%–17%)
Work as a volunteer without pay	491 (9%, 8%–10%)	421 (9%, 8%–10%)
Have a primary paid job	894 (16%, 15%–17%)	1,097 (23%, 22%–24%)^b^

McNemar test.

^a^ p = 0.001.

^b^ p < 0.0001. Data show frequency (%, 95%CI).

In 2018, men participated in fewer physical and leisure activities, besides decreasing their participation at religious services, contrary to women who continue with these activities and increase their participation as family caregiver. Notably, 794/2,121 (37%) of men have found a paid employment again in 2018, compared to only 451/2,572 (18%) in 2012 (dependent proportion difference p < 0.0001) (Table 3).

**TABLE 3:** Prevalence of advanced activities of daily living by year stratify by gender. From 2012 to 2018 MHAS data.

Activity	2012		2018	
	Women (n = 3,014)	Men (n = 2,572)	Women (n = 2,616)	Men (n = 2,121)
Attending a sports or social club	198 (7%, 6%–8%)	195 (8%, 7%–9%)	141 (5%, 4%–6%)^a^	134 (6%, 5%–7%)^b^
Attend a lecture, seminar, or class	437 (15%, 14%–16%)	376 (15%, 14%–16%)	249 (10%, 9%–11%)	155 (7%, 6%–8%)^c^
Sew, embroider, knit or other crafts	1,124 (37%, 36%–38%)	580 (23%. 22%–24%)^d^	1,044 (40%, 37%–41%)	102 (5%, 4%–6%)^d^ ^e^
Attend religious services	2,532 (84%, 83%–85%)	2,083 (81%, 80%–82%)^c^	2,204 (84%, 83%–85%)	1,582 (75%, 74%–76%)^d^ ^e^
Talk to relatives or friends on the phone or use the internetetc.	2,155 (72%, 71%–73%)	1,744 (68%, 67%–69%)^c^	2,138 (82%, 81–83)^e^	1,565 (74%, 73%–75%)^d^ ^e^
Take care of a sick family member	493 (16%, 15%–17%)	418 (16%, 15%–17%)	536 (21%, 20%–22%)^f^	295 (14%, 13%–15%)^d^ ^b^
Participate in any volunteer work in community	500 (17%, 16%–18%)	502 (20%, 19%–21%)^c^	466 (18%, 17%–19%)	310 (15%, 14%–16%)^c^ ^e^
Work as a volunteer without pay	238 (8%, 7%–9%)	249 (10%, 9%–11%)	220 (8%, 7%–9%)	201 (10%, 9%–11%)
Have a primary paid job	443 (15%, 14%–16%)	451 (18%, 17%–19%)^c^	303 (12%, 11%–13%)^f^	794 (37%, 36%–38%)^d^ ^c^
≥3 AADLs performed	1,555 (52%, 50%–53%)	1,193 (46%, 44%–48%)^d^	1,386 (53%, 51%–55%)	891 (42%, 40%–44%)^d^ ^c^

McNemar test 2012 vs 2018.

^a^ p< 0.01.

^b^ p< 0.05.

^c^ p < 0.01. Chi-squared test women vs men.

^d^ p < 0.0001. Chi-squared test women vs men.

^e^ p< 0.0001. McNemar test 2012 vs 2018.

^f^ p = 0.001. Data show frequency (%, 95%CI).

Age is a confounding factor in the performance of AADLs; therefore, we conducted a double stratification using the cut-off value of life expectancy in Mexico (75 years) in 2012 and 2018. We observed that the tendency of execution of AADLs did not change in men and women; thus, performing these activities is independent of age itself (Table 4).

**TABLE 4:** Prevalence of AADLs by year stratify by gender and age group (cut-off 75 years). From 2012 to 2018 MHAS data.

Activity	2012				2018				
	Women (n = 3,014)^a^		Men (n = 2,572)^b^		Women (n = 2,616)^c^		Men (n = 2,121)^d^		
	<75 years (n = 2,462)	≥75 years (n = 550)	<75 years (n = 2063)	≥75 years (n = 508)	<75 years (n = 1,582)	≥75 years (n = 1,031)	<75 years (n = 1,251)		≥75 years (n = 867)
Attending a sports or social club	171 (7% 6%–8%)	27 (5% 4%–6%)	162 (8% 7%–9%)	33 (7% 6%–8%)	91 (6% 5%–7%)	50 (5% 4%–6%)	93 (7% 6%–8%)		41 (5% 4%–6%)
Attend a lecture, seminar, or class	380 (15% 14%–16%)	58 (11% 10%–12%)	315 (15% 14%–16%)	61 (12% 11%–13%)	175 (11% 10%–12%)^e^	73 (7% 6%–8%)^e^	114 (9% 8%–10%)^e^		40 (5% 4%–6%)^f^ ^e^
Sew, embroider, knit or other crafts	915 (37% 36%–38%)	208 (39% 38%–40%)	511 (25% 24%–26%)^g^	69 (14% 13%–15%)^g^	672 (43% 42%–44%)^e^	371 (36% 35%–37%)	71 (6% 5%–7%)^g^ ^e^		30 (4% 3%–5%)^g^ ^e^
Attend religious services	2,073 (84% 83%–85%)	459 (84% 83%–85%)	1,690 (82% 81%–83%)^f^	393 (77% 76%–78%)^f^	1,347 (85% 84%–86%)	852 (83% 82%–84%)	926 (74% 73%–75%)^g^ ^e^		655 (75% 74%–76%)^g^ ^e^
Talk to relatives or friends on the phone or use the internetetc.	1,796 (73% 72%–74%)	359 (65% 64%–66%)	1,441 (70% 69%–71%)^f^	303 (60% 59%–61%)	1,321 (84% 83%–85%)^e^	814 (79% 78%–80%)^e^	968 (77% 76%–78%)^g^ ^e^		596 (69% 68%–70%)^g^ ^e^
Take care of a sick family member	440 (18% 17%–19%)	53 (10% 9%–11%)	353 (17% 16%–18%)	65 (13% 12%–14%)	361 (23% 22%–24%)^h^	163 (16% 15%–17%)^i^	199 (16% 15%–17%)^g^		91 (11% 10%–12%)^j^
Participate in any volunteer work in community	418 (18% 17%–19%)	70 (13% 12%–14%)	408 (21% 20%–22%)^k^	68 (14% 13%–15%)	313 (20% 19%–21%)	152 (15% 14%–16%)	214 (17% 16%–18%)^e^		95 (11% 10%–12%)^f^ ^e^
Work as a volunteer without pay	211 (9% 8%–10%)	28 (5% 4%–6%)	209 (10% 9%–11%)	41 (8% 7%–9%)^l^	148 (9% 8%–10%)	71 (7% 6%–8%)	136 (11% 10%–12%)		65 (8% 7%–9%)
Have a primary paid job	3,833 (16% 15%–17%)	60 (11% 10%–12%)	371 (18% 17%–19%)^f^	80 (16% 15%–17%)^f^	229 (15% 14%–16%)	74 (7% 6%–8%)^e^	579 (46% 45%–47%)^g^ ^e^		215 (25% 24%–26%)^g^ ^e^

^a^ 2 missing data.

^b^ 1 missing data.

^c^ 3 missing data.

^d^ 3 missing data. Data show frequency (%, 95%CI).

^e^ p< 0.0001. Comparison between the years 2012-2018 McNemar test.

^f^ p< 0.05. Comparison between genders by age group, chi-squared test.

^g^ p < 0.0001.

^h^ p< 0.01. Comparison between the years 2012-2018 McNemar test.

^i^ p< 0.05.

^j^ p = 0.001. Comparison between genders by age group, chi-squared test.

^k^ p < 0.01.

^l^ p = 0.05.

### 3.3 Risk factors to performance <3 AADLs

The multi-comparison models included statistically significant factors such as male gender, age group, urban residency, schooling, stroke, cognitive impairment, and sedentary lifestyle. After controlling for these confounding factors, the risk of not performing ≥3 AADLs was in overall model HR = 1.25 (95%CI: 1.17–1.37), women HR = 1.20 (95%CI: 1.08–1.32), and men HR = 1.26 (95%CI: 1.17–1.35), all statistically significant (HRs p < 0.0001). In all probed models, the proportional-hazards assumptions were held, and the survey clustering was handled.

**FIGURE 4:**
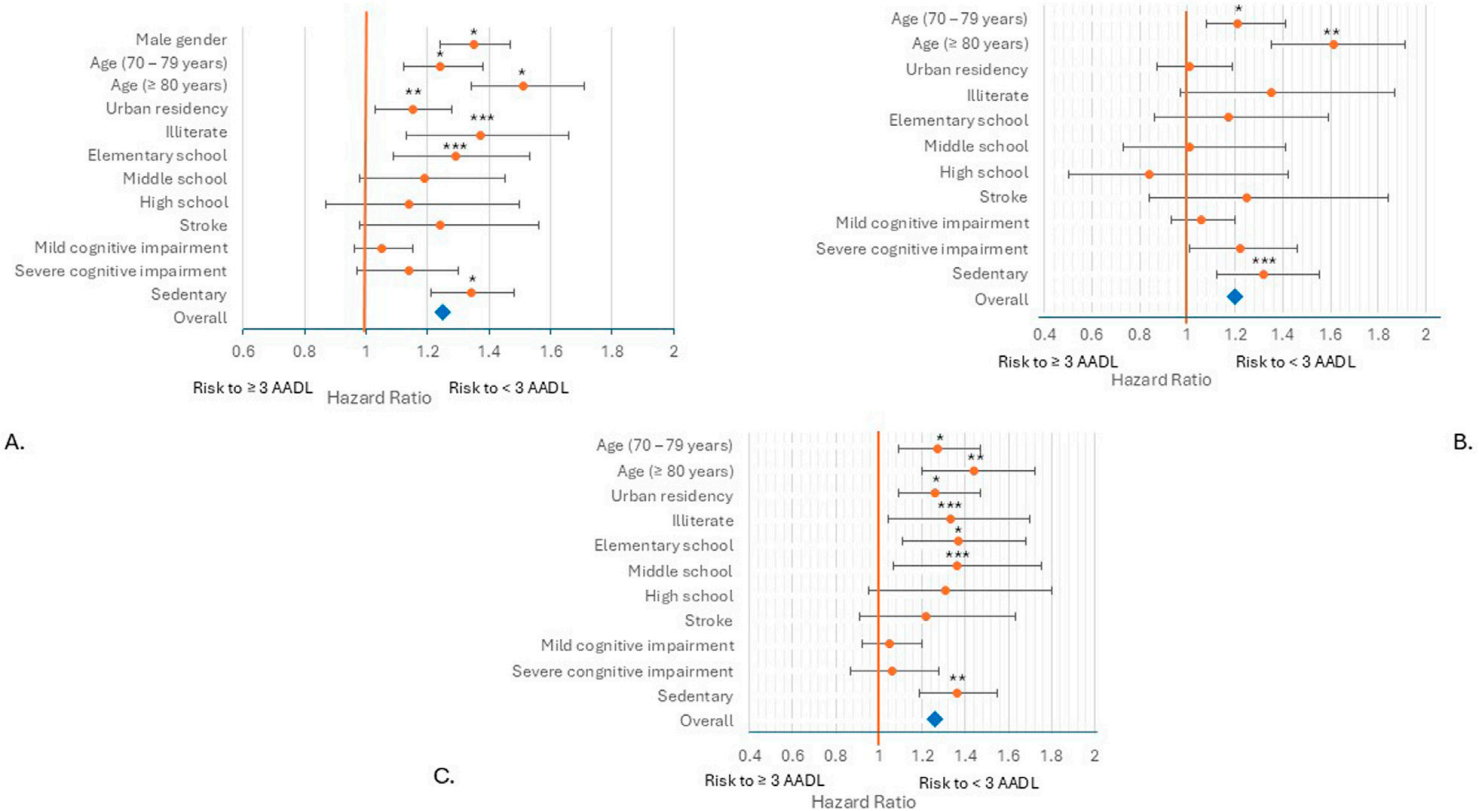
Risk factors to not perform ≥3 AADLs. Cox regression (p < 0.0001) in all models. **(A)** Overall model HR = 1.25, 95%CI: 1.17–1.37, *p < 0.0001; **p < 0.05; ***p ≤ 0.001. **(B)** Women model HR = 1.20, 95%CI: 1.08–1.32, *p < 0.01; **p < 0.0001; ***p = 0.001. **(C)** Men model HR = 1.26, 95%CI: 1.17–1.35, *p < 0.01; **p < 0.0001; ***p < 0.05.

Overall model shows that age >80 years (HR = 1.51, 95%CI: 1.34–1.71, p < 0.0001), illiterate subject (HR = 1.37, 95%CI: 1.13–1.66, p = 0.001), male gender (HR = 1.34, 95%CI: 1.24–1.47, p < 0.0001), and physical activity <3 times/week (HR = 1.34, 95%CI: 1.21–1.48, p < 0.0001) were the main risk factors to not performing ≥3 AADLs. Other risk factors were age between 70 and 79 years, and if the subject had only elementary school education (Figure 4A). Separating by gender in women only the following factors—age over 70 years and sedentary lifestyle—did not warrant the performance of ≥3 AADLs (Figure 4B). However, in men, age over 70 years, low scholarship, living in an urban location and sedentary lifestyle were risk factor for not performing ≥3 AADLs (Figure 4C).

**FIGURE 5:**
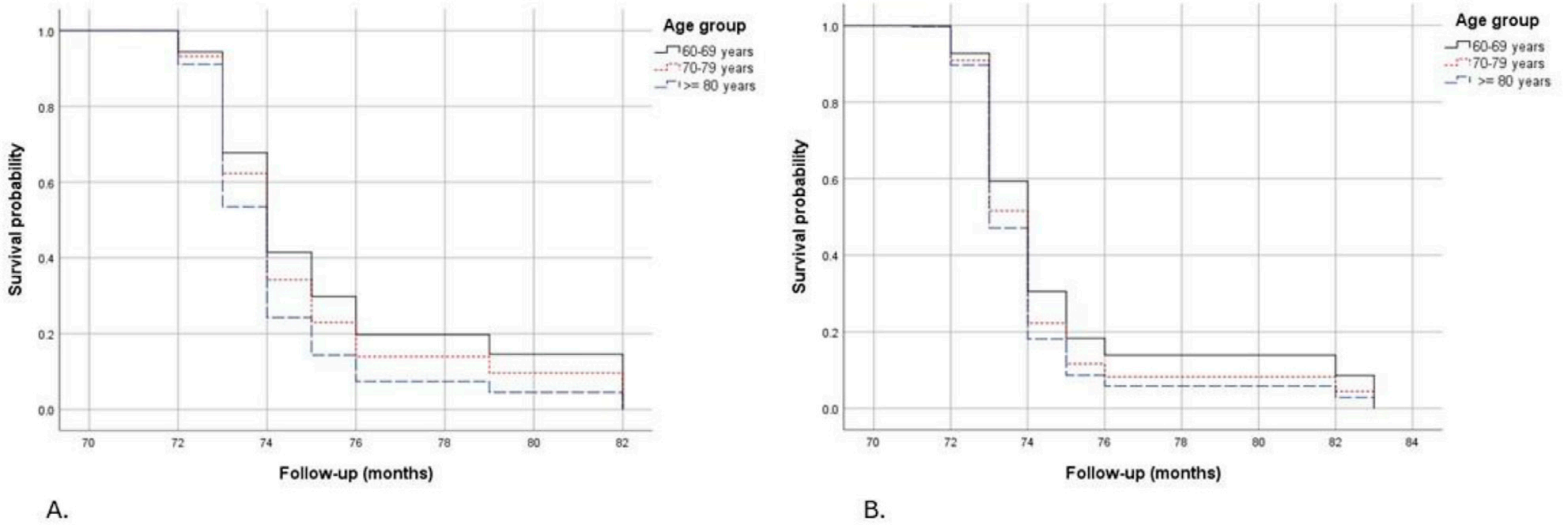
Kaplan-Meier survival plots to not perform ≥3 AADLs controlled by sociodemographic, lifestyle and health conditions factors, separated by gender and stratified by age group. **(A)** Women, **(B)** Men.

Survival plots show the difference between women and men in carrying out ≥3 AADLs. Women between 60 and 79 years were more likely to perform ≥3 AADLs than men, but at 80 years or more, both genders had similar possibilities (Figures 5A,B); therefore, the execution of 3 or more AADLs is age-dependent over 80 years; but before 80 years, this capacity is gender-dependent.

## 4 Discussion

In this work, we found a decrease in the total AADLs carried out in 2018 compared to 2012 MHAS waves (2.68 ± 1.39 vs 2.61 ± 1.34, p < 0.01), although women performed more AADLs in 2018 than in 2012, contrary to men. In addition, the risk factors to not perform ≥3 AADLs are different in women and men. The risk factors for women are age ≥70 years and sedentary lifestyle, and men have those factors in addition to low scholarship and live in urban locations. After control by confounder factors, the risk of not performing ≥3 AADLs was in the overall model HR = 1.25 (95%CI: 1.17–1.37), women HR = 1.20 (95%CI: 1.08–1.32), and men HR = 1.26 (95%CI: 1.17–1.35). To our knowledge, this is the first study that shows a sex difference of AADLs performance with a population base and large sample size.

As we previously pointed out, there is still no agreement on how to measure HA, but the different proposals agree that the inclusion of social activities, as well as issues of health and lifestyle, are part of this construct (Behr et al., 2023; McLaughlin et al., 2020; Fauzi et al., 2024). We propose that the assessment of AADLs represents the FC and involves IC, because of the complexity of their execution. In this sense, it is important to recognize that an older adult who has a controlled NCDs may have adequate FC and can perform AADLs without inconvenience.

In this work we have found that after 6 years of follow-up, 48% of the community-dwelling older adults could still perform ≥3 AADLs; however, there is a small decrease in the average of activities. Previously, we showed that the execution of AADLs depends on several factors such as age, gender, education level, urban location and sedentary lifestyle in a transversal study (Sánchez-Rodríguez et al., 2023). Now we observed the same factors in a longitudinal study, could highlight differences between women and men.

Independent of their health condition, between 52% and 53% of women perform ≥3 AADLs compared to 42% of men, which is also observed in Chilean older adults reporting that 54% of women can carried out more than 2 AADLs (de Albuquerque Araújo et al., 2024), and 55% of Brazilian aged women (Mazer et al., 2024). While women are biologically more vulnerable and tend to experience more NCDs, they have a long-life expectancy (Fritz et al., 2024; Talifu et al., 2024; Rueda-Salazar et al., 2021), as was observed in this work. They maintained their FC by engaging in a complex series of activities, such as AADLs, that involve both basic and instrumental activities (Cornelis et al., 2019; Talifu et al., 2024). An essential component of AADLs is social activity, that is, the interrelation with other people in any environment, which appears to be influenced by the social conditions of the country and culture. In high developed countries such as United States of America, women have higher rates of HA with higher social activity (2.7%–6.3% more HA than man) (Chu et al., 2023); while in Singapore there was no gender difference in active life expectancy (Chan et al., 2016), being an indirect form to assess AADLs. On the other hand, in South Korea the women with lower social activity are less healthy (-9.3% to -10.9%) because they have less social pastime activities than men (71% vs 76%) (Chu et al., 2023; Kim and Choi, 2024); and the Costa Rican women without social participation have lower healthy years expectancy compared with men and their Spain and Chilean counterparts (Rueda-Salazar et al., 2021; Chu et al., 2023).

In Mexico, women from this cohort are generally responsible for family care and household activities, as well as being more social and religious, with active participation in the community, which is evident in this work. Mexican women may have adapted positively to daily adversity, that is, they have developed resilience. In this context, it was reported that resilience is increased in women with an adaptation to stress and with social activities, such as those of Hispanic origin (Springfield et al., 2022) because the resilience influences directly the AADLs (Martino et al., 2025). In the last stage of life, maintaining the performance of AADLs and social participation could potentially prevent frailty (Costenoble et al., 2023).

After 6 years of follow-up, there has been a shift in both genders’ activities, with a decrease in attendance at academic classes or performing crafts and an increase in computer and internet use. Between 2012 and 2018, several technological changes worldwide favored digital interpersonal communication, particularly among this age group, which enabled them to connect with their family and friends, preventing potential social isolation. Several means of communication became available, supporting sending and receiving text messages, voice calls, photos, and videos. Applications such as WhatsApp have been widely used in Mexico since 2014 (Baulch et al., 2020). The use of internet and smartphones has increased over the years and older adults recognize digital skills and their usefulness for daily lives, which is an important issue of HA (Gallo et al., 2024). Regarding this, different authors who pooled a nationally representative sample of older adults from five longitudinal studies, including the MHAS, CHARLS (China), ELSA (England), HRS (United States), and SHARE (Europe), concluded that digital exclusion of elders could lead to functional dependence, cognitive impairment, and frailty (Gallo et al., 2024; Lu X. et al., 2022; Wang et al., 2024; Li, 2024). Therefore, older adults increasing their phone/computer and internet use in 6 years was a positive aspect toward HA.

However, social isolation encompasses not only digital exclusion but also the absence of participation in community or religious activities (Kammar-García et al., 2023). In this respect, the participation rate of women did not change over time but that of men decreased. That is, older Mexican women were more socially integrated, increasing their capacity to perform more AADLs. Like older women in the United States, they were more likely to develop a HA (Chu et al., 2023; Li et al., 2018) and well-being (Das and Dhillon, 2024). Moreover, it is possible to live longer, because social isolation is a risk factor for all-cause mortality in Mexicans elders (Kammar-García et al., 2023).

In addition, we found that men returned to paid work, probably because of economic need. In recent years, far from public policies, Mexico has opened opportunities for semi-informal employment for this age group. Although the occupations are not gender-specific, women tend to be engaged in supportive activities for a parent or relative, reducing the available number of hours to be employed or the likelihood of accessing a full-time job (Stampini et al., 2022).

Consequently, the risk factors for not performing ≥3 AADLs are different between men and women. Notably, there are fewer risk factors for women than for men, for women only age over 70 years and a sedentary lifestyle prevent them from performing AADLs, whereas for men, being over 70 years and having a sedentary lifestyle, in addition to low schooling and urban residency were the risk factors. Although our study population has poor schooling independent of gender, men’s activities are most affected. In this sense, low education and unfavorable environment have an impact on cognitive health and the possibility of HA (McLaughlin et al., 2020; Das and Dhillon, 2024; Lu C. et al., 2022); however, these factors are not related to the possibility of performing AADLs in women.

In the case of sedentary lifestyle, there is no difference by gender. The importance of retirement in determining physical activity behaviours in old age has been recognized, but this is only applicable if older adults have a job. A systematic review points out that physical activity and leisure-time increases after the retirement transition (Barnett et al., 2012), which is discordant with our results because the study population—mainly women—performs limited physical activities, but this is unfavorable for the execution of AADLs in men. Although the benefit of changing a sedentary lifestyle to light or moderate physical activity increases the odds of HA (Shi et al., 2024), there has been resistance to adopting this lifestyle change.

The last risk factor is age. As an individual age, their physical deterioration becomes greater; thus, there are limitations to performing AADLs. However, we find that the difference by gender is high, because women continue to perform these activities at older ages. Nevertheless, at 80 years old, few older adults can perform ≥3 AADLs, regardless of their gender. In an individual, AADLs loss after the eighth decade of life increases the risk of transitioning from pre-fragility to fragility, but women can regain strength through social involvement (Costenoble et al., 2023). This continues to be controversial because a study with the MHAS cohort to analyze factors associated with HA in older adults aged above 70 years shows that being a woman is a negative factor for HA from 77 to 90 years. This discrepancy with our results is because of the extant HA concept, as these authors included only subjects without NCDs and cognitive impairment (Arroyo-Quiroz et al., 2020). Thus, this concept of HA seems somewhat restrictive. Indeed, as we noted above, women tend to experience NCDs more frequently, but this does not prevent them from performing more AADLs.

Finally, in this study we conducted a secondary analysis of databases previously obtained by people outside our research group, therefore we do not have control of the data, which is a limitation. Another limitation is that the survey was self-reported, which prevents the confirmation of the information. Furthermore, the AADLs were selected from the questions of the survey, and although this construct was previously validated, it has modest reliability (KR-20 = 0.60) (Sánchez-Rodríguez et al., 2023); thus, more activities could have been taken into consideration. Moreover, it is possible to have a survivor bias from 6 years attrition in the cohort.

Despite these limitations, the study has several strengths such as being a representative national sample with a large sample size, including both rural and urban areas. Furthermore, the study covers 6 years and highlights significant world changes, particularly technological ones, that older adults had to adapt to, as we observed.

In addition, this is the first study to demonstrate gender differences in the performance of complex tasks, specifically focused on social, leisurely and productive activities for older individual adults living in community settings, without or with mild cognitive impairment, even if they have any NCD.

In conclusion, our findings suggest that women have a greater capacity to adapt to social changes associated with aging, allowing them to perform ≥3 AADLs than men. Furthermore, factors associated with not performing ≥3 AADLs in women include age ≥70 years and a sedentary lifestyle. Men lose this capacity at age 70 if they have low education and live in urban areas, in addition to a sedentary lifestyle. However, it is important to note that in most Latin American countries, such as Mexico, community-level gerontological programs have a feminist orientation due to the fact that the majority of participants are women. This could be a determining factor in the relative advantages of women in performing a greater number of AADLs. For this reason, programs and public policies must be implemented to strengthen a favorable environment, promoting the development of inclusive generative projects for men and women, so that they have reasons to continue pursuing AADLs for as long as possible in their lives. Longitudinal studies with primary data are needed to confirm our findings.

## Data Availability

The original contributions presented in the study are included in the article/Supplementary Material, further inquiries can be directed to the corresponding author.
