# Supplementary material for: Gender differences in advanced activities of daily living: evidence from the longitudinal study of health and aging in Mexico 2012–2018
Source: Front Aging. 2025 Jul 28;6:1544493. doi: 10.3389/fragi.2025.1544493 (PMC12336443; doi:10.3389/fragi.2025.1544493)
Supplement: Supplementary file 1 [file Table2.docx]

**Table 1.** Advanced activities of daily living of the available questions of the Mexican Health and Aging Study 2012 and 2018 waves (Sánchez-Rodríguez et al, 2023 (11)).

| Physical/leisure domain | Social domain | Productive domain |
| --- | --- | --- |
| Attend to a sport or social club | Do you attend religious services? | In the last 2 years, did you participate in any volunteer work for a religious, educational, charity organization or for the community? |
| Attend to a lecture, seminar, or class | Talk to relatives or friends on the phone with or use the computer to send email or use the Internet | Work as a volunteer or help with a non-profit organization without pay or compensation. |
| Sew, embroider, knit or other crafts | Did you take care of a sick family member? | During the last year, did you have a primary paid job? |
